# Dose distribution of intensity-modulated proton therapy with and without a multi-leaf collimator for the treatment of maxillary sinus cancer: a comparative effectiveness study

**DOI:** 10.1186/s13014-019-1405-y

**Published:** 2019-11-21

**Authors:** Soichi Sugiyama, Kuniaki Katsui, Yuki Tominaga, Takahiro Waki, Norihisa Katayama, Hidenobu Matsuzaki, Shin Kariya, Masahiro Kuroda, Kazunori Nishizaki, Susumu Kanazawa

**Affiliations:** 1 Departments of Radiology, Dentistry and Pharmaceutical Science, Okayama University Graduate School of Medicine, 2-5-1 Shikata-cho, Kita-ku, Okayama, 700-8558 Japan; 2 Department of Radiology, Tsuyama Chuo Hospital, Tusyama, Okayama 708-0841 Japan; 3 Departments of Proton Beam Therapy, Dentistry and Pharmaceutical Science, Okayama University Graduate School of Medicine, 2-5-1 Shikata-cho, Kita-ku, Okayama, 700-8558 Japan; 4 Department of Radiation Technology, Tsuyama Chuo Hospital, Tusyama, Okayama 708-0841 Japan; 5 Departments of Radiology, Okayama University Hospital, 2-5-1 Shikata-cho, Kita-ku, Okayama, 700-8558 Japan; 6 Departments of Oral Diagnosis and Dentomaxillofacial Radiology, Okayama University Hospital, 2-5-1 Shikata-cho, Kita-ku, Okayama, 700-8558 Japan; 7 Departments of Otolaryngology-Head and Neck Surgery, Dentistry and Pharmaceutical Science, Okayama University Graduate School of Medicine, 2-5-1 Shikata-cho, Kita-ku, Okayama, 700-8558 Japan; 8 Department of Radiological Technology, Graduate School of Health Sciences, Okayama University, 2-5-1 Shikata-cho, Kita-ku, Okayama, 700-8558 Japan

**Keywords:** Multi-leaf collimator, Chemoradiotherapy, Intensity-modulated proton therapy, Pencil beam scanning, Maxillary sinus cancer

## Abstract

**Background:**

Severe complications, such as eye damage and dysfunciton of salivary glands, have been reported after radiotherapy among patients with head and neck cancer. Complications such as visual impairment have also been reported after proton therapy with pencil beam scanning (PBS). In the case of PBS, collimation can sharpen the penumbra towards surrounding normal tissue in the low energy region of the proton beam. In the current study, we examined how much the dose to the normal tissue was reduced by when intensity-modulated proton therapy (IMPT) was performed using a multi-leaf collimator (MLC) for patients with maxillary sinus cancer.

**Methods:**

Computed tomography findings of 26 consecutive patients who received photon therapy at Okayama University Hospital were used in this study. We compared D2% of the region of interest (ROI; ROI-_D2%_) and the mean dose of ROI (ROI-_mean_) with and without the use of an MLC. The organs at risk (OARs) were the posterior retina, lacrimal gland, eyeball, and parotid gland. IMPT was performed for all patients. The spot size was approximately 5–6 mm at the isocenter. The collimator margin was calculated by enlarging the maximum outline of the target from the beam’s eye view and setting the margin to 6 mm. All plans were optimized with the same parameters.

**Results:**

The mean of ROI-_D2%_ for the ipsilateral optic nerve was significantly reduced by 0.48 Gy, and the mean of ROI-_mean_ for the ipsilateral optic nerve was significantly reduced by 1.04 Gy. The mean of ROI-_mean_ to the optic chiasm was significantly reduced by 0.70 Gy. The dose to most OARs and the planning at risk volumes were also reduced.

**Conclusions:**

Compared with the plan involving IMPT without an MLC, in the dose plan involving IMPT using an MLC for maxillary sinus cancer, the dose to the optic nerve and optic chiasm were significantly reduced, as measured by the ROI-_D2%_ and the ROI-_mean_. These findings demonstrate that the use of an MLC during IMPT for maxillary sinus cancer may be useful for preserving vision and preventing complications.

## Background

Cancers of the nasal cavity and paranasal sinuses are uncommon and comprise about approximately 3–5% of all head and neck cancers. Standard treatment methods for locally advanced sinonasal carcinomas include surgical resection and adjuvant radiation therapy [1, 2]. In addition, preoperative chemoradiotherapy (CRT) has been successful in achieving a negative surgical margin [3]. At the Okayama University Hospital, preoperative CRT is performed for T3–4 large maxillary sinus tumors, with a total dose of 66 Gy. However, after conventional irradiation, three-dimensional radiation therapy, and intensity-modulated radiation therapy (IMRT), serious (grade 3–4) complications, such as eye damage and salivary gland dysfunction, have been reported at a rate of 1–24% [4–6]. In addition, the use of proton beam, heavy particle beam therapy, or intensity-modulated proton therapy (IMPT)—instead of three-dimensional radiation therapy—reduces the rate of complications and improves the local control rate [7].

Treatment with proton beam therapy involves two types of irradiation methods: passive scattering proton therapy and IMPT, which is among the more recently developed irradiation methods. To cover a target during IMPT, each beam is scanned laterally across the target using magnetic fields in a technique called pencil beam scanning (PBS). Similar to IMRT, PBS enables state-of-the-art IMPT that optimizes all beams to deliver a sufficient dose to the target. However, serious adverse events such as visual impairment have also been reported after treatment with proton beams using PBS for head and neck cancers [8]. On the other hand, during PBS, collimation can reduce the penumbra of the surrounding normal tissue in the low energy region of the proton beam [9, 10]. Winterhalter et al. referred to the possibility of improving the dose distribution in the head region using a multi-leaf collimator (MLC) [11]. In addition, Moignier et al. reported that IMPT for head tumors improves the marginal dose while using clinical data [12]. Moreover, Yasui et al. showed that the dose distribution of peripheral organs at risk (OARs) was improved when IMPT was administered via an aperture, even in the region including the head and neck area [13]. However, their study was not limited to a single site of tumor disease. To the best of our knowledge, no previous study has examined the extent by which the dose to the normal tissue is reduced by when IMPT with an MLC is used for a single tumor site in the head and neck region. Therefore, in this study, we examined by how much the dose to the normal tissue was reduced by when IMPT with an MLC is used for maxillary sinus cancer.

## Methods

### Patients and consent

The imaging results of 26 consecutive patients undergoing photon therapy at the Okayama University Hospital between September 2009 and March 2017 were used in this study. All subjects were simulated in the preoperative setting for irradiation and had undergone arterial infusion chemotherapy for maxillary sinus carcinoma, with the exception of 3 patients in each treatment category (Table 1). Patients provided written informed consent for undergoing treatment and were provided the option to opt out of this study via notifications displayed in the outpatient ward and on the Okayama University Hospital’s website. The presiding institutional review board approved this study (approval number 1712–011).

**Table 1:** Demographic, clinical, and treatment characteristics of patients (*n* = 26)

Characteristic	N(%)
Gender	
Female	5(19%)
Male	21(81%)
Maxillary sinus	
Right	13(50%)
Left	13(50%)
Histological type	
Squamous cell carcinoma	24(92%)
Spindle cell carcinoma	1(4%)
Small cell carcinoma	1(4%)
T stage	
T2	2(8%)
T3	11(42%)
T4	13(50%)
N stage	
N-	26(100%)
N+	0(0%)
Radiotherapy	
Preoperative	23(88%)
Definitive	3(12%)
Chemotherapy	
No	0(0%)
Yes	
Arterial infusion chemotherapy	23(88%)
Other chemotherapy	3(12%)

### Target and OARs

Planning computed tomography (CT) scans acquired in the treatment planning position with 2-mm sliced were transferred to MIM Maestro, ver. 6.6.7 (Cleveland, Ohio US) for delineation of the gross tumor volume (GTV) and OARs and for the planning at risk volumes (PRVs). The GTV and OARs were contoured by a radiation oncologist, with confirmation from two additional specialists in radiation oncology. A margin of 3 mm was added to the GTV to calculate the clinical target volume (CTV). The CTV was then corrected for natural anatomic boundaries such as the bone or air cavities. Delineated OARs included the brainstem, optic chiasm, pituitary gland, optic nerve, posterior retina, lacrimal gland, eyeballs, parotid gland, and cochlea. A 3-mm margin was added to the brainstem, optic chiasm, optic nerve, and cochlea to obtain the respective PRVs [14, 15]. PRVs were not modified even if the CTV and PRV overlapped.

### Robustness

The value of 3 mm was the margin of uncertainty for patients with head and neck tumors, allowing for machine variability. The value of 3.5% was the range of uncertainty resulting from uncertainties in the range calculation, the acquisition of CT number, and the CT number-stopping power conversion table [13, 16].

### Treatment planning and dose prescription

The pencil beam scanning method was used for all patients undergoing proton therapy using the system at our institute (Hitachi, Tokyo, Japan). The proton therapy system was equipped with an MLC. All IMPT treatment planning was performed on the RayStation, version 7 (Raysearch Laboratories AB, Stockholm, Sweden). For all patients, planning was performed with 2 coplanar beams and 1 non-coplanar beam [17]. The same gantry angle was used for both sides. For right side tumors, the gantry angles of coplanar beams were set at 0° and 90°, and the non-coplanar gantry angle was 45°. For left side tumors, the gantry angles of coplanar beams were set at 0° and 270°, and the non-coplanar beam angle was 315°. The radiation therapy dose to the CTV before surgery for maxillary sinus cancer was 66 Gy in 33 fractions. Dose computations used a proton pencil beam model with optimization for intensity-modulated proton fields. The dose constraints used were based on previous reports (Table 2) [18].

**Table 2:** Dose constraints and recommendations for intracranial organs at risk when conventional fractionation is used

OAR and PRV	Constraints
Optic chiasm	Dmax < 54 Gy
Optic nerve	Dmax < 54 Gy
Pituitary gland	Dmax < 50 Gy
Brainstem	Dmax < 54 Gy
Retina	Dmax < 45 Gy
Eyeball	Dmax < 45 Gy
Lens	Dmax < 6 Gy
Lacrimal gland	V30 Gy < 50%
Cochlea	Dmean 45 Gy

*OAR* organ at risk, *PRV* planning organ at risk volume

### Spot size and margin of an MLC

An MLC margin was required to assure the marginal dose of the target. As the spot spacing was affected by spot size, the spot size was approximately 5–6 mm at the isocenter. The collimator margin was computed by expanding the maximum outline of the target from the beam’s eye view with the margin set to 6 mm [13].

### Optimization parameters

All plans were optimized with the same parameters to evaluate differences due to IMPT with or without an MLC, i.e., the same constrained optimization functions for CTV, OARs, and PRV objectives with the related parameters, the same gantry for with and without an MLC, as well as the same parameters, as shown in Table 3. To emphasize the differences in the MLC, the applied constraints in this study were those that were not chosen during generation of the clinical treatment plans.

**Table 3:** Parameters

ROI name	Description	Weight	Robust
CTV	Min DVH 6600 cGy to 95% volume	50	+
CTV	Max DVH 7042 cGy to 10% volume	10	–
CTV	Max DVH 7920 cGy to 2% volume	10	–
CTV	Min DVH 6138 cGy to 98% volume	10	–
Optic chiasm + 3 mm	Max DVH 5400 cGy to 2% volume	10	–
Optic chiasm	Max DVH 5400 cGy to 2% volume	5	–
Pituitary gland	Max DVH 4500 cGy to 2% volume	1	–
Brainstem + 3 mm	Max DVH 5000 cGy to 2% volume	5	–
Brainstem	Max DVH 5000 cGy to 2% volume	2	–
Posterior retina in both eyes	Max DVH 4500 cGy to 2% volume	5	–
Parotid gland on both sides	Max EUD 3000 cGy	1	–
Optic nerve in both eyes + 3 mm	Max DVH 5400 cGy to 2% volume	5	–
Optic nerve in both eyes	Max DVH 5400 cGy to 2% volume	10	–
Lens in both eyes	Max DVH 600 cGy to 2% volume	1	–
Lachrymal gland in both eyes	Max EUD 3000 cGy	5	–
Both eyeballs	Max DVH 4500 cGy to 2% volume	3	–
Cochlea on both sides	Max DVH 4500 cGy to 2% volume	3	–
Cochlea on both sides + 3 mm	Max DVH 4500 cGy to 2% volume	1	–
Body contour	Max DVH 7042 cGy to 2% volume	5	–

*ROI* region of interest, *CTV* clinical target volume, *DVH* dose volume histogram, *EUD* equivalent uniform dose, *Max* maximum, *Min* minimum

### Plan evaluation

We compared the dose indexes for plans with and without an MLC. We prepared both plans with equal CTV D95% doses. We examined the relative change of some dose indexes such as the D2% of the region of interest (ROI; ROI-_D2%_) and the mean doses of the ROI (ROI-_mean_) of the OARs, PRVs, and irradiation volume (V5 Gy, V10 Gy, V15 Gy, V20 Gy, and V25 Gy of the ipsilateral lacrimal gland).

### Statistical analysis

We analyzed individual dose volume histograms for both treatment plans using Wilcoxon rank sum tests, with *p* < 0.05 considered statistically significant. Statistical analyses were performed using SPSS software, version 22 (IBM, NY, USA).

## Results

**Fig. 1:**
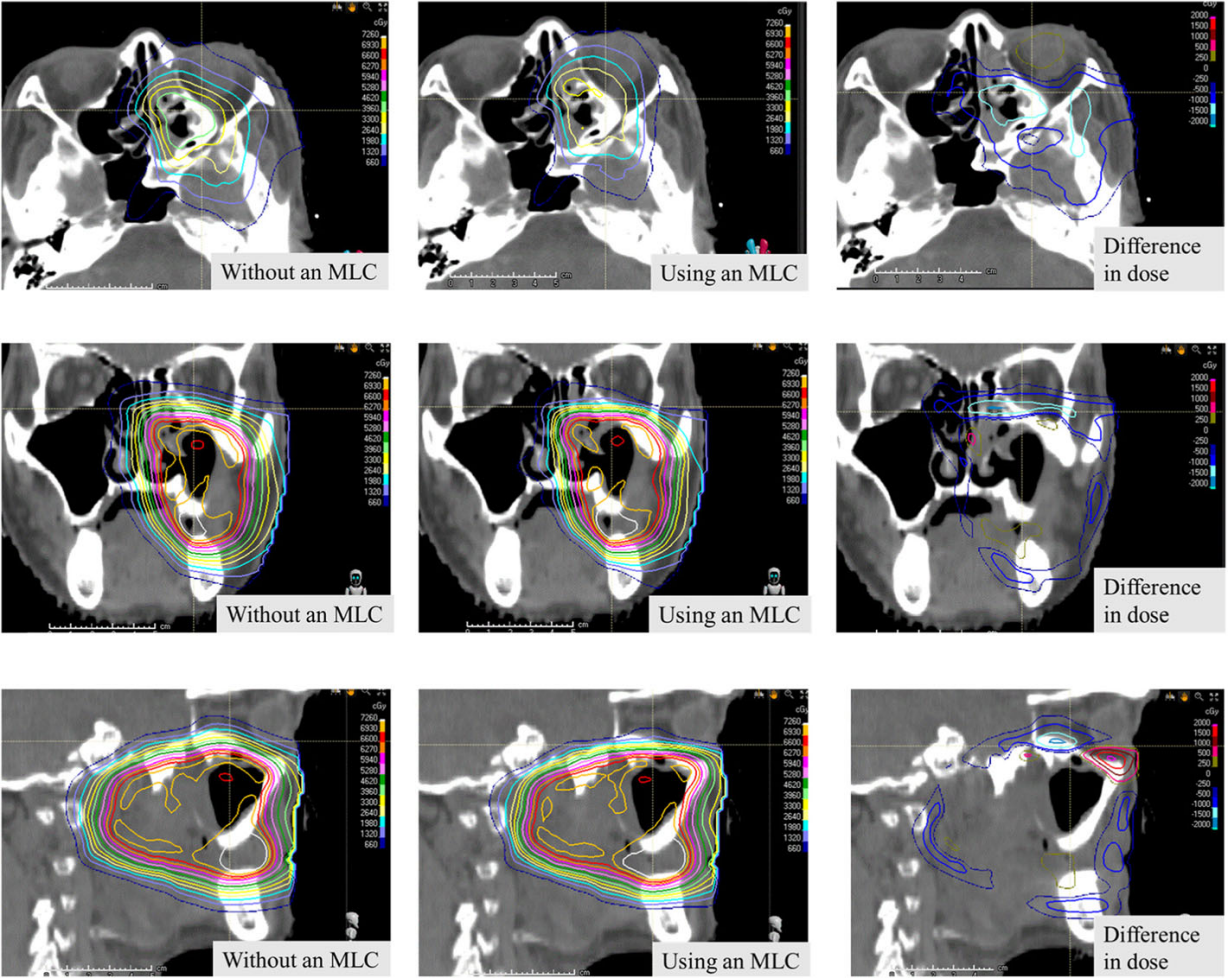
Views of the dose distributions during the treatment of maxillary sinus cancer. MLC, multi-leaf collimator

**Fig. 2:**
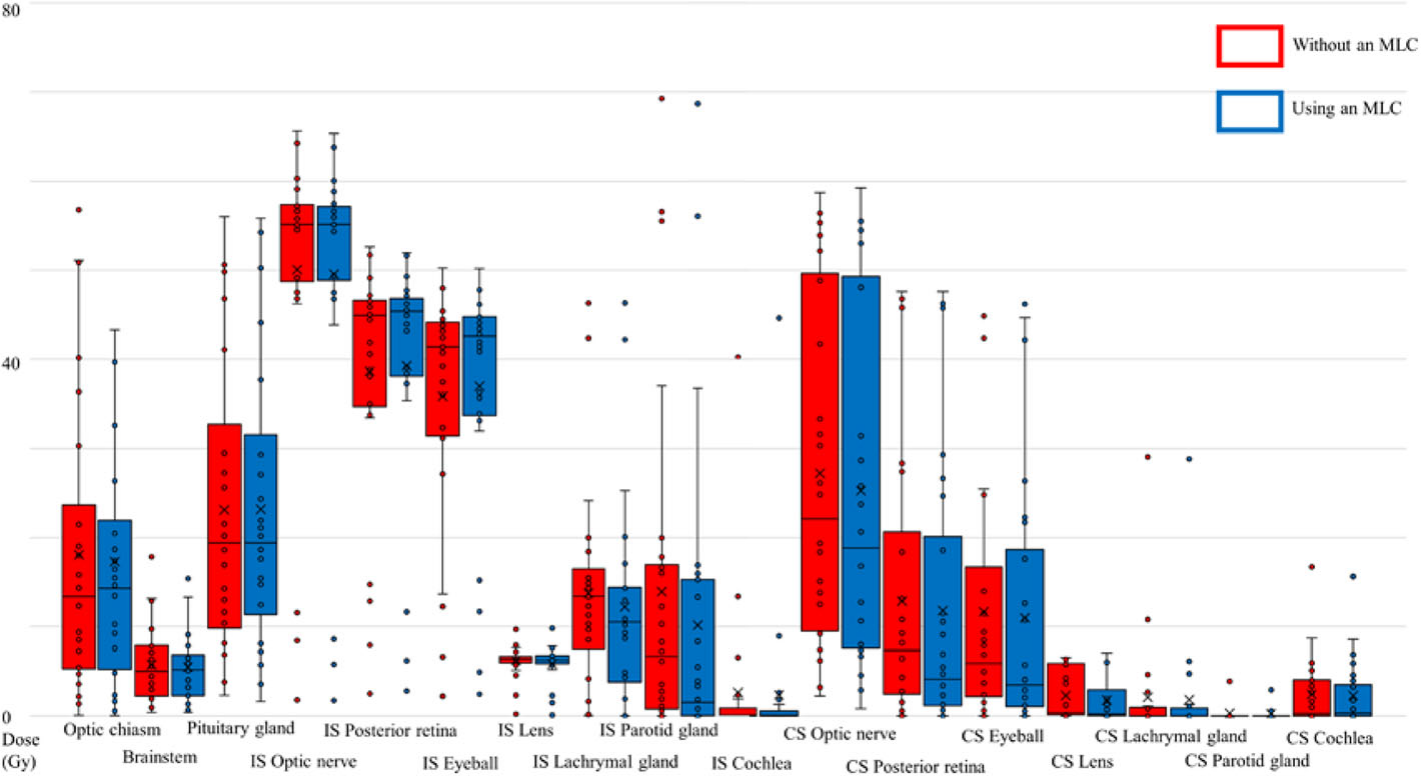
Box and dot plots of OARs and PRVs by ROI-_D2%_. ROI-_D2%,_ D2% of the region of interest; MLC, multi-leaf collimator; OAR: organ at risk; PRV: planning at risk volume; IS, ipsilateral side; CS, contralateral side

**Fig. 3:**
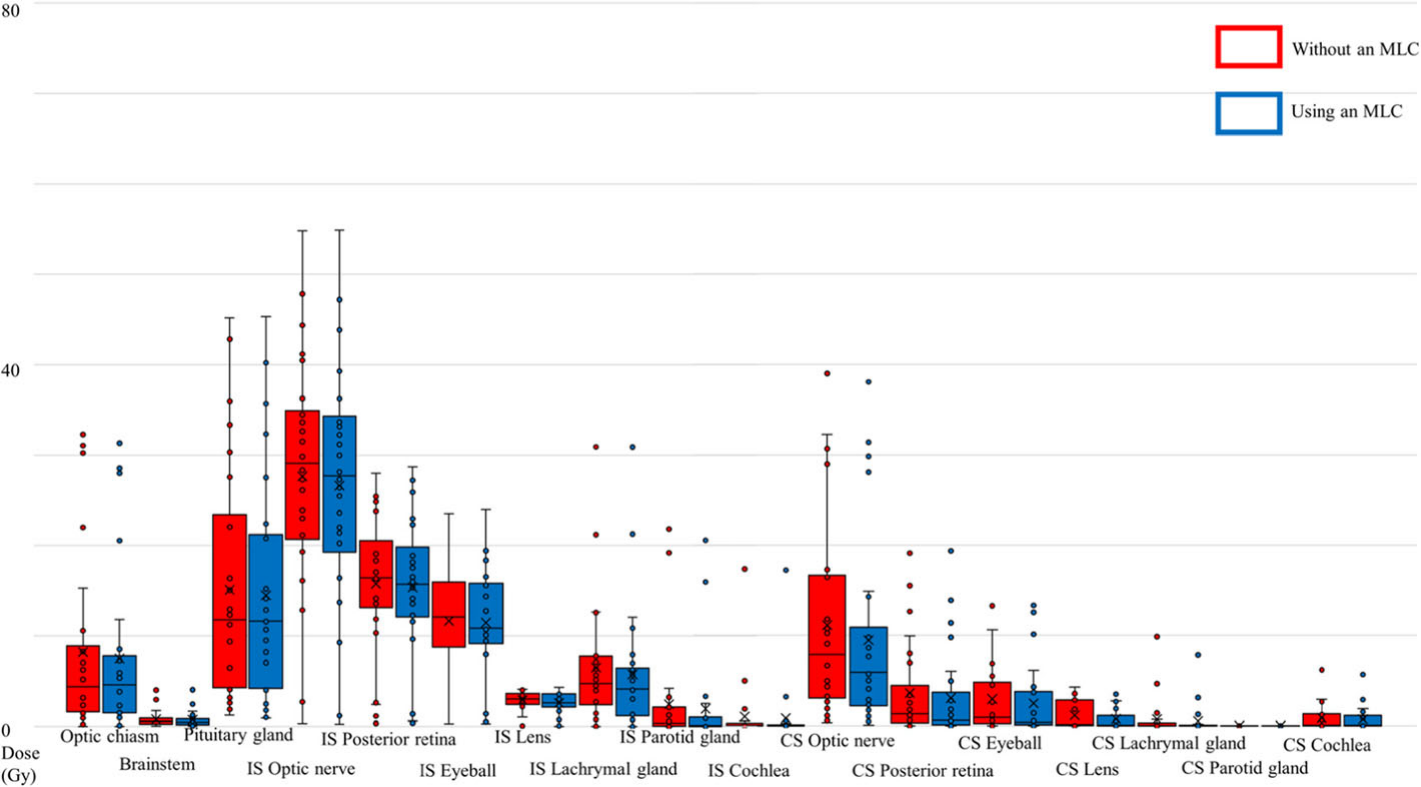
Box and dot plots of OARs and PRVs by ROI-_mean_. ROI-_mean,_ mean dose of region of interest dose; MLC, multi-leaf collimator; OAR: organ at risk; PRV: planning at risk volume; IS, ipsilateral side; CS, contralateral side

Typical dose distribution and its difference in dose planning with and without an MLC are shown in Fig. 1. The IMPT plan using an MLC resulted in a sharp lateral penumbra, and the dose to the ipsilateral optic nerve was reduced. The OARs and PRV dose metrics are shown in Figs. 2 and 3 as boxplots for each patient overlaid with boxplots summarizing the data. The central colored line in the boxplots represents the median, with the edges representing the 25th and 75th percentiles. The whiskers show the range of data excluding outliers. The central, dashed black line represents the mean. Wilcoxon signed rank tests were performed between each pair of treatment modalities.

The parameters of OARs and PRVs in dose planning with or without an MLC are shown in Table 4. As shown in Table 4, the mean of ROI-_D2%_ and ROI-_mean_ of the ipsilateral optic nerve were significantly reduced in the group with an MLC. The mean of ROI-_mean_ of the optic chiasm was also significantly reduced in the MLC group, as was the mean of ROI-_D2%_ of the ipsilateral optic nerve, which reduced from 50.06 Gy to 49.58 Gy, with a difference of 0.48 Gy. Similarly, the mean of ROI-_mean_ of the ipsilateral optic nerve was reduced from 27.66 Gy to 26.62 Gy, with a difference of 1.04 Gy. In the optic chiasm, the mean of ROI-_mean_ was reduced from 8.20 Gy to 7.50 Gy, indicating a difference of 0.70 Gy. Other OARs and PRVs are shown in Table 4. A dose reduction was observed in most OARs and PRVs, with the exception of the D2% of the optic chiasm, pituitary gland, ipsilateral posterior retina, and eyeball, as well as the ROI-_mean_ of the ipsilateral posterior retina, ipsilateral eyeball, and contralateral parotid gland.

**Table 4:** ROI-_D2%_, ROI-_mean,_ and difference in OARs and PRVs in IMPT planning without and with an MLC

		Mean (Gy)	Range (Gy)	Difference (Gy)	*p* value	* *p*<0.05			Mean (Gy)	Range (Gy)	Difference (Gy)	*p* value	* *p*<0.05
ROI-_D2%_, difference (without an MLC - using an MLC) ROI-_D2%_, difference (without an MLC - using an MLC)													
Optic chiasm	MLC-	18.04	56.8-0.08	0.78	0.096		Brainstem	MLC-	5.75	17.82-0.39	0.27	0.034	*
	MLC+	17.27	56.52-0.06					MLC+	5.48	15.65-0.4			
Pituitary gland	MLC-	23.13	56.03-2.31	-0.05	0.99								
	MLC+	23.19	55.86-1.63										
IS Optic nerve	MLC-	50.06	65.59-1.80	0.48	0.016	*	CS Optic nerve	MLC-	27.21	58.71-2.23	1.92	0	*
	MLC+	49.58	65.35-1.75					MLC+	25.29	59.27-0.82			
IS Posterior retina	MLC-	38.63	52.64-2.51	-0.63	0.049	*	CS Posterior retina	MLC-	12.84	47.61-0	1.05	0	*
	MLC+	39.27	51.95-2.81					MLC+	11.79	47.61-0.01			
IS Eyeball	MLC-	35.86	50.25-2.23	-1.1	0.003	*	CS Eyeball	MLC-	11.65	44.99-0	0.67	0.005	*
	MLC+	36.95	50.18-2.46					MLC+	10.98	46.19-0.01			
IS Lens	MLC-	6.23	9.92-0.22	0.38	0.006	*	CS Lens	MLC-	2.29	6.54-0	0.51	0	*
	MLC+	5.85	9.85-0.11					MLC+	1.79	7.06-0			
IS Lacrimal gland	MLC-	13.73	46.29-0.05	1.5	0.001	*	CS lacrimal gland	MLC-	2.1	29.07-0	0.41	0.005	*
	MLC+	12.22	46.32-0.01					MLC+	1.69	28.84-0			
IS Parotid gland	MLC-	13.91	69.24-0	3.76	0	*	CS Parotid gland	MLC-	0.32	3.89-0	0.07	0.345	*
	MLC+	10.16	68.66-0					MLC+	0.25	2.93-0			
IS Cochlea	MLC-	2.65	40.22-0	0.27	0.02	*	CS Cochlea	MLC-	2.45	16.69-0	0.14	0.044	*
	MLC+	2.38	44.62-0					MLC+	2.31	15.62-0			
ROI-_mean_, difference (without an MLC - using an MLC) ROI-_mean_, difference (without an MLC - using an MLC)													
Optic chiasm	MLC-	8.2	32.29-0.02	0.7	0	*	Brainstem	MLC-	0.82	4.01-0.04	0.09	0	*
	MLC+	7.5	31.29-0.01					MLC+	0.73	4.04-0.04			
Pituitary gland	MLC-	15.1	45.19-1.25	0.62	0.038	*							
	MLC+	14.49	45.35-0.94										
IS Optic nerve	MLC-	27.66	54.82-0.35	1.04	0	*	CS Optic nerve	MLC-	11.11	38.99-0.36	1.66	0	*
	MLC+	26.62	54.88-0.25					MLC+	9.45	38.06-0.10			
IS Posterior retina	MLC-	15.77	28.02-0.35	0.41	0.134		CS Posterior retina	MLC-	3.61	19.11-0	0.54	0	*
	MLC+	15.36	28.70-0.39					MLC+	3.07	19.35-0			
IS Eyeball	MLC-	11.65	23.55-0.28	0.2	0.282		CS Eyeball	MLC-	2.96	13.31-0	0.48	0	*
	MLC+	11.45	23.99-0.29					MLC+	2.48	13.31-0			
IS Lens	MLC-	2.91	4.09-0.07	0.31	0.004	*	CS Lens	MLC-	1.16	4.27-0	0.39	0	*
	MLC+	2.6	4.30-0.03					MLC+	0.78	3.49-0			
IS Lacrimal gland	MLC-	6.49	30.92-0.01	0.75	0.001	*	CS Lacrimal gland	MLC-	0.75	9.84-0	0.23	0.008	*
	MLC+	5.75	30.89-0					MLC+	0.51	7.81-0			
IS Parotid gland	MLC-	2.43	21.85-0	0.48	0	*	CS Parotid gland	MLC-	0.02	0.32-0	0.02	0.068	
	MLC+	1.95	20.58-0					MLC+	0.01	0.18-0			
IS Cochlea	MLC-	1.02	17.35-0	0.16	0.006	*	CS Cochlea	MLC-	0.87	6.17-0	0.13	0.008	*
	MLC+	0.86	17.22-0					MLC+	0.74	5.66-0			

*ROI* region of interest, *ROI-*_*D2%*_ D2% of region of interest, *ROI-*_*mean*_ mean doses of the region of interest, *OAR* organ at risk, *PRV* planning organ at risk volume, *MLC* multi-leaf collimator, *IS* ipsilateral side, *CS* contralateral side

* *p* < 0.05 Significant difference

**Fig. 4:**
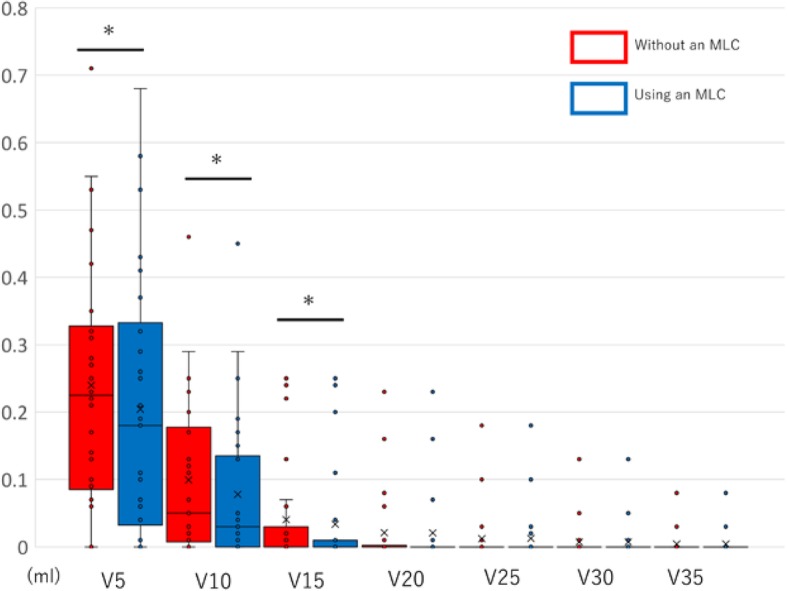
Box and dot plots of the ipsilateral lacrimal gland dose volume metrics. * ; *p* < 0.05 Significant difference

As shown in Fig. 4, the irradiation volumes of the lacrimal gland with and without an MLC were as follows: V5 Gy, 0.23 cm^3^ vs. 0.20 cm^3^, difference = 0.03 cm^3^, *p* = 0.080; V10 Gy, 0.11 cm^3^ vs. 0.08 cm^3^, difference = 0.03 cm^3^, *p* = 0.003; and V15 Gy, 0.05 cm^3^ vs. 0.20 cm^3^, difference = 0.01 cm^3^, *p* = 0.007. In general, these data show significant reductions associated with the use of an MLC in the low dose area only, while no significant differences were found above V20 Gy.

The beam energy was automatically selected by the treatment planning device and was 70.70–126.50 MeV. The mean beam monitor unit (MU) used in the plan was 21,82,193/fraction (range, 12,30,730–38,85,439) without an MLC and 23,34,309/fraction (range, 13,53,181–40,76,774) with an MLC. The use of an MLC resulted in an increase in the MU in all cases, increasing on average by 7% (range, 2–17%).

## Discussion

The results of the current study demonstrate that, compared to IMPT without an MLC, IMPT using an MLC resulted in a significantly lower the mean of ROI-_D2%_ to the ipsilateral optic nerve, as well as lower the mean of ROI-_mean_ to the ipsilateral optic nerve and optic chiasm, while providing an optimal dose to the treatment targets for maxillary sinus cancer.

Although postoperative CRT is the standard treatment for T3–4 maxillary sinus cancer, there are also reports of inoperable cases, surgical refusal, and the use of preoperative CRT [4, 6, 19]. Multidisciplinary treatment with surgery, chemotherapy, and radiation therapy is performed for maxillary sinus cancer [2, 3]. Visual impairments have been reported as a complication of surgery and photon therapy for maxillary sinus cancer on both the ipsilateral and contralateral sides [19]. In recent years, particle beam therapy has been attempted for paranasal sinus cancers to reduced sever complications. However, Fukumitsu et al. reported brain necrosis and optic dysfunction, even with proton beam therapy for sinus cancer [8]. During proton beam treatment for ethmoid sinus cancer, which is relatively similar to maxillary sinus cancer, adverse events include optic neuropathy and cerebral necrosis [20]. Thus, dose reduction to normal organs is necessary to avoid complications such as optic neuropathy and cerebral necrosis.

On phantom experiments, a reduction in penumbra due to low energy regional collimation with PBS was reported [21]. In the current study, the beam energy was 70.70–126.50 MeV. We planned to use low energy and the use of an MLC should have improved the penumbra more clearly, and the marginal dose was better and the dose distribution was improved.

In current study, the dose reductions were small with and without the use of an MLC. OARs and PRVs dose in PBS proton therapy will depend on the parameters used for optimization. These include patient distance, tumor depth, irradiation angle, PBS beam quality, algorithms for optimization of treatment planning systems, and the use of optimization and an MLC. The cases included in our study were consecutive cases of maxillary sinus cancer at the Okayama University Hospital. Also, the size, degree of invasion, and shape of each tumor are different, all plans are prepared under the same conditions, except for the use of an MLC, to eliminate planning bias. We speculate that the plan under this condition was the reason for the small difference of our result. Better dose distribution may be achieved if IMPT constraint optimization and gantry angle are fully considered.

According to Yasui et al. [13], the use of an MLC for lesions in the head and neck region has led to cases where the dose to the optic nerve decreased by a few percent and the dose to the optic chiasm decreased by up to 32%. However, that study was not focused on a single disease and the evaluated OARs were different for each irradiation site. In contrast, the current study showed that the dose to the optic nerve and lacrimal gland can be reduced using an MLC during IMPT among patients with a single disease. In the overall comparison of the mean ROI-_D2%_ and ROI-_mean_ with and without an MLC, the mean of ROI-_D2%_ for the ipsilateral optic nerve was reduced by 0.48 Gy and the mean of ROI-_mean_ was reduced by 1.04 Gy. The mean of ROI-_mean_ to the optic chiasm was reduced by 0.70 Gy.

In previous studies regarding the effect of radiation on the lacrimal gland, no significant dry eye symptoms were observed in patients who received a mean dose of < 30 Gy to the lacrimal gland during photon radiation therapy and these studies have shown symptoms in the all patients treated with doses > 45 Gy and with doses > 57 Gy during photon radiation therapy [22, 23]. Although the lacrimal gland did not receive a high enough dose to cause complications when the mean doses were administered with and without an MLC (5.7 Gy and 6.5 Gy, respectively) in the current study, the dose of lacrimal gland was decreased significantly. This finding may be useful when irradiation is performed near the lacrimal gland.

Yasui et al. showed the utility of the patient-specific aperture system. In their study, the OAR dose was reduced by several percent to several tens of percent owing to the patient-specific aperture system [13]. We believe this was the reason why the dose reduction effect to the surrounding OAR was low in our study compared to that in the study by Yasui et al. First, it is possible that the spot size used in our treatment system was smaller (5–6 mm at the isocenter) than that of their machine, which was 7.2–11.8 mm at the isocenter. According to Moteabbed et al., a smaller spot size is associated with a less effective MLC [24]. Second, in the current study, PRVs were not modified even if the CTV and PRV overlapped to prevent planned bias, which was observed in several plans. Third, to prevent bias owing to the plan, the gantry angle and dose constraints were fixed in all plans.

In the current study, we used the collimating MLC technique along the largest edge of the tumor. Daniel et al. reported a dynamic collimation system in which collimation is performed by moving two blades per layer with the beam spot [25, 26]. In their method, collimation can be performed on all layers of the tumor. The penumbra of the beam on the distal side and on the proximal side of the tumor is reduced; it is thought that the concentration of the proton beam is improved. Therefore, the dose distribution of the surrounding normal tissue is significantly improved by approximately 13.65% compared to cases that were treated without the dynamic collimation system [12]. Further advances in therapeutic devices may show potential for improved dose distribution.

Even if an MLC is used, there are no additional costs for each patient. In contrast, the use of MLC plans increased the MU by mean of 7% (range, 2–17%). Therefore, the increase in MU slightly increases the treatment time. The dose of the ipsilateral eyeball was increased by the use of an MLC. Doses to the eye and surrounding normal tissue are issues of optimization and gantry angle, suggesting that optimization and examination of angles are necessary for each case.

This study has some limitations, including the relatively small sample size of 26 cases. Moreover, it was not possible to obtain MR fusion images, and we did not re-plan treatments as might be performed during adaptive therapy. Finally, the dose calculation algorithm used in this study did not include Monte-Carlo simulations.

## Conclusion

During IMPT, the use of an MLC for the treatment of maxillary sinus cancer reduces the dose to the ipsilateral optic nerve. Also, the dose of most OARs and PRVs were reduced in the MLC group. In the future, a large-scale prospective study should be conducted to determine the effectiveness of the collimation, the results of which can be used to improve IMPT dose distribution and thus reduce adverse events.

## Data Availability

The data will not be shared because the ethics committees did not allow sharing of the data.

## Abbreviations

CRT: Chemoradiotherapy
CT: Computed tomography
DVH: Dose volume histogram
IMPT: Intensity-modulated proton therapy
IMRT: Intensity-modulated radiation therapy
MLC: Multi-leaf collimator
OAR: Organ at risk
PBS: Pencil beam scanning
PRV: Planning at risk volume
PTV: Planning target volume
